# Solar Water Disinfection to Produce Safe Drinking Water: A Review of Parameters, Enhancements, and Modelling Approaches to Make SODIS Faster and Safer

**DOI:** 10.3390/molecules26113431

**Published:** 2021-06-05

**Authors:** Ángela García-Gil, Rafael A. García-Muñoz, Kevin G. McGuigan, Javier Marugán

**Affiliations:** 1 Department of Chemical and Environmental Technology (ESCET), Universidad Rey Juan Carlos, C/Tulipán s/n, Móstoles, 28933 Madrid, Spain; angela.garcia.gil@urjc.es (Á.G.-G.); rafael.garcia@urjc.es (R.A.G.-M.); 2 Department of Physiology & Medical Physics, RCSI University of Medicine and Health Sciences, DO2 YN77 Dublin, Ireland; kmcguigan@rcsi.ie

**Keywords:** large-volume containers, SODIS materials, kinetic model, weathering, radiation distribution, sensitisers

## Abstract

Solar water disinfection (SODIS) is one the cheapest and most suitable treatments to produce safe drinking water at the household level in resource-poor settings. This review introduces the main parameters that influence the SODIS process and how new enhancements and modelling approaches can overcome some of the current drawbacks that limit its widespread adoption. Increasing the container volume can decrease the recontamination risk caused by handling several 2 L bottles. Using container materials other than polyethylene terephthalate (PET) significantly increases the efficiency of inactivation of viruses and protozoa. In addition, an overestimation of the solar exposure time is usually recommended since the process success is often influenced by many factors beyond the control of the SODIS-user. The development of accurate kinetic models is crucial for ensuring the production of safe drinking water. This work attempts to review the relevant knowledge about the impact of the SODIS variables and the techniques used to develop kinetic models described in the literature. In addition to the type and concentration of pathogens in the untreated water, an ideal kinetic model should consider all critical factors affecting the efficiency of the process, such as intensity, spectral distribution of the solar radiation, container-wall transmission spectra, ageing of the SODIS reactor material, and chemical composition of the water, since the substances in the water can play a critical role as radiation attenuators and/or sensitisers triggering the inactivation process.

## 1. Introduction

Water is a precious but scarce resource that is essential for life. Only 3.5% of the Earth’s water is freshwater and fit for human consumption and of this freshwater, only 1% is free flowing in rivers, lakes, or streams. Even so, there is sufficient drinking water on the planet to meet the needs of the population. However, accessibility and availability of safe drinking water (free from pathogens and priority chemical contamination) at the household level is not equal for all. Ensuring these basic needs is one of the greatest challenges currently facing humanity.

On 28 July 2010, the United Nations (UN) General Assembly recognised the human right to water and sanitation, declaring that clean drinking water and sanitation are essential to fulfil all human rights [1]. In September 2015, the same assembly announced the Sustainable Development Goals (SDGs) in the 2030 Agenda action plan. There are 17 SDGs and all work towards “ending poverty in all its forms”. They are the successors of the Millennium Development Goals (MDG) signed in 2000, but these new goals incorporate a specific sixth SDG on water. SDG6 aims to “Ensure availability and sustainable management of water and sanitation for all” and includes eight global targets related to management of natural water resources, wastewater, and the environment. The first target is to “Achieve access to safe and affordable drinking water” before 2030 since water accessibility is not guaranteed for a significant 29% of humanity. For example, 844 million people still lack access to safe drinking water, and approximately 1000 children die each day due to diseases related to unsafe drinking water or sanitation [2,3]. Furthermore, availability of water is becoming more unreliable and problematic due to the effects of the climate crisis [4,5,6], the water demand of an ever-increasing population, the expansion of cities, and the developing economy [7,8].

The lack of safe drinking water affects communities in low-to-medium-income countries most. For example, in sub-Saharan Africa, one in every ten children under 5 years old dies due to diarrhoea [9]. The lack of financial and technological resources impedes the implementation of drinking water treatment plants in these regions. To deal with this situation, Household Water Treatments (HWT) are required, since they tend to be [10]:

- Low-cost: The most impoverished communities are the most affected.
- User-friendly: Everybody should easily produce safe drinking water.
- Sustainable: To avoid the need for consumables that are expensive or hard to obtain.

Most HWT are small-scale adapted water treatments that can be used by anyone. They can also include pre-treatments to remove solids that negatively influence disinfection treatment. At the household level, very simple forms of pre-treatment can be provided, including filtration through fabric or sand filters, flocculation-coagulation with natural substances, or sedimentation. However, these pre-treatments do not completely eliminate the pathogenic microorganisms (bacteria, viruses, and protozoa) responsible for waterborne diseases and other health risks in low-income countries [11,12].

**Figure 1:**
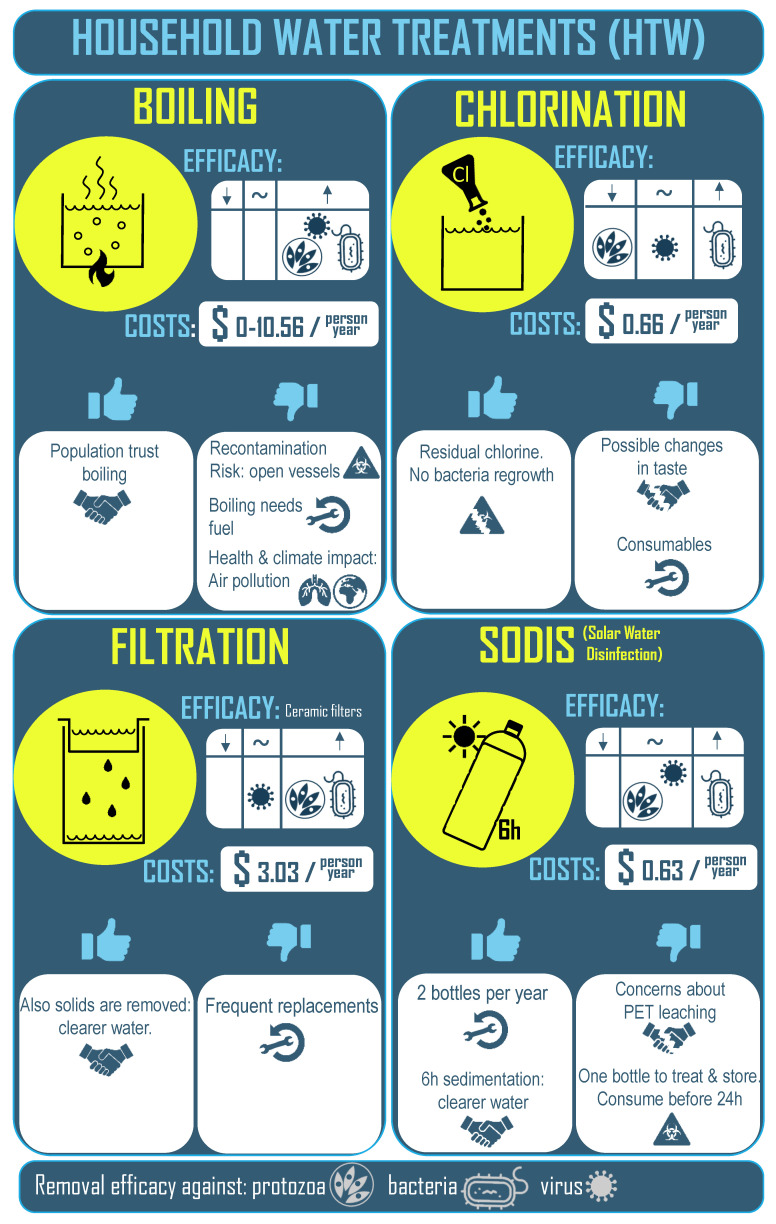
Household water treatments.

The most widely adopted HWT for pathogen removal are the following, and their characteristics are summarised in Figure 1:

- Boiling: This process is highly effective against all classes of microbial pathogens [13]. People universally accept that boiling makes water safer to drink, so they trust the treatment and adopt it readily. Boiling requires large amounts of fuel with estimated costs of up to $10.56 per person per year [14], unless it is freely collected (in the form of firewood. However, boiling causes health risks due to indoor air pollution and boiled water is very vulnerable to recontamination since it usually is cooled in open containers [11,15].
- Chlorination: Chlorine can be applied in liquid or tablet form, is also easy to apply at household level, and is very inexpensive (estimated costs of $0.66 per person per year) [16]. A particular strength of chlorine is that residual chlorine in the water can protect against bacterial regrowth. However, chlorine is less effective against some viruses and ineffective against common protozoa. Disinfection by-products can be formed due to reactions with naturally occurring substances. These by-products can change the smell and taste of the treated water. Chlorination is sometimes rejected on these grounds. Furthermore, heavy use requires consumables that must be replaced periodically, and, in general, the population that demands HWT is in difficult to access, remote areas [12,17].
- Filtration: Generally, filters do not remove all pathogens since their filter pores are larger than the microorganisms. However, ceramic filters do retain protozoa, work well against bacteria, and some of them have efficacy against viruses (the smallest pathogen). Users have confidence in filters since they tend to clarify the water, and ceramic filters can also evaporatively cool the water [12,18]. Nevertheless, ceramic filters are fragile and maintenance can be expensive (estimated costs of $3.03 per person per year) [16].
- SODIS: Solar water disinfection, or SODIS, is based on the germicidal effect of UV light and its synergistic effect with rise in water temperature. The procedure is very user-friendly since it involves just filling a transparent container with water and placing it in direct sunlight for several hours. The treatment is cheap because only a transparent container such as a glass or plastic container is required (estimated costs of $0.63 per person per year) [16]. Generally, UVA radiation from the sun is lethal against bacteria, as is UVB radiation against bacteria, viruses, and protozoa [19,20,21]. No adverse effect on the water’s taste has been observed. However, it is recommended that the treated water is consumed within the 24 h following exposure since bacteria can regrow in the dark while the water is stored and cooling. Water turbidity decreases the solar disinfection efficiency and prolongs treatment time. An additional benefit is that since the water is generally treated and then stored in the same container, there is a decreased risk of recontamination [22].

SODIS has been found to be one of the most appropriate treatments for producing safe drinking water, because it is inexpensive and not dependent on consumables. The SODIS process is driven entirely by solar energy, and its effectiveness for the removal of pathogens from water has been widely proved. The most widely accepted procedure for this simple technology is described in detail in the “*SODIS manual: Guidance on solar water disinfection*” published by Luzi et al., (2016) [23]. Briefly, water with a maximum level of 30 NTU should be exposed to the sunlight in clean 2-litre polyethylene terephthalate (PET) bottles for 6 h on sunny days, 48 h on cloudy days, while on days of continuous rainfall, SODIS should not be used. PET bottles are selected due to their low-cost and wide availability. Concerns about chemical contaminants from plastic migration have been addressed by previous studies [24,25,26]. SODIS has been accepted by the World Health Organisation (WHO) and has been recommended for low-income countries and in the aftermath of natural disasters or humanitarian crises [27,28]. The implementation of SODIS treatment requires behaviour changes that sometimes generates obstacles to uptake. In this sense, any new SODIS-based innovations should be user-friendly, supported by local community elders, and ergonomically designed for a favourable reception [10]. Many studies have reported successful SODIS implementation under diverse field conditions in locations such as Kenya, Cameroon, India, Cambodia, and Latin America [29,30,31,32].

However, standard 2-litre PET bottle-based SODIS presents some obstacles to widespread adoption. The low volume (2 L) of the most available bottles can necessitate the use of up 25 bottles per family to ensure provision of the daily water requirement estimated by the WHO (50–100 L per person and day). The risk of recontamination risk increases as the number of bottles in use increases. Furthermore, while the effectiveness of SODIS has been excellent against bacterial waterborne pathogens of concern, it is limited against some viruses and protozoa. PET plastic does not transmit UVB radiation, so the inactivation of viruses and protozoa significantly slows down or is, in some cases, negligible. The variability of weather conditions and of the water characteristics necessitates the incorporation of an additional safety cushion into the standard recommended exposure time from 6 h on sunny days to up to 48 h in cloudy conditions. These three obstacles can all be addressed if the volume and material of the SODIS containers are revised and if the kinetic models that forecast the required solar exposure time, accurately considers all the involved variables. The aim of this work is to summarise the current knowledge of the variables affecting SODIS efficacy and review the developed kinetic models in order to make the SODIS process safer and faster.

## 2. SODIS: Variables

SODIS guidance has been published to facilitate a standard procedure for worldwide implementation. However, this general method has limitations. Several variables must be exhaustively studied since they interfere with radiation transfer from sunlight to the pathogen and, consequently, determine the treatment efficiency.

### 2.1. Radiation

It is well-known that the higher the radiation intensity, the higher the cell damage. However, the photoinactivation mechanism, and, consequently, the inactivation rate, varies strongly with wavelength.

#### 2.1.1. Photoinactivation Mechanisms

Microorganisms are photoinactivated when they suffer damage triggered by an excited chromophore (any substance capable of absorbing photons). Photoinactivation can be conducted via direct or indirect damage.

Direct damage is an endogenous process that occurs when photon absorption by a chromophore induces changes to the chemical structure. The chromophore is generally a constituent of the microorganism’s genome (e.g., nucleic acids, proteins, or other macromolecules). Since all pathogens have a genome, all of them are susceptible to this type of damage.

In the case of indirect damage, photon absorption by a chromophore produces photo-produced reactive intermediates (PPRI) that damage components of the microorganism. In this instance, the chromophore is called a sensitiser. Depending on the location of the sensitiser, indirect photoinativation can be exogenous or endogenous [21].

Endogenous indirect inactivation takes place when PPRI are generated from internal sensitisers. Examples of internal sensitisers are amino acids, coenzymes, vitamins, or metalloproteins that mainly produce reactive oxygen species (ROS) such as hydrogen peroxide, hydroxyl radicals, singlet oxygen, or superoxide radicals. Only microorganisms with sufficient internal sensitisers are subject to this type of damage.

Exogenous indirect inactivation happens when the sensitiser is external, such as dissolved organic matter, nitrates, nitrites, or metal complexes. Depending on water quality, diverse PPRI can be externally formed, e.g., ROS, carbonate radicals (CO_3_^•−^) [33], or reactive halogen species (RHS) in seawater [34]. This mechanism is only possible if the extra-cellular water matrix contains these sensitisers. Therefore, in pure water, exogenous indirect inactivation does not occur.

#### 2.1.2. Solar Spectrum

Different portions of the solar spectrum participate in the three mechanisms of photoinactivation. This wavelength dependence stems from the different chromophores with different sensitivities and absorption spectra that are involved [35].

(Endogenous) direct damage: Photons in the UVB range (280–320 nm) mainly contribute to (endogenous) direct damage since RNA and DNA absorption spectra extend up to 320 nm [35,36,37]. UVB wavelengths are more energetic than UVA radiation. Despite the UVB radiation intensity at the Earth’s surface is relatively low, it can trigger harmful damage, which is more than sufficient to kill biological cells.

Endogenous indirect damage: This damage is primarily initiated by UVB, UVA, and visible (400–700 nm) photons. Internal components of some microorganisms such as coenzymes, vitamins, and metaloproteins can generate internal PPRI when they are illuminated with UVA and UVB radiation. Flavins and porphyrins can also be activated with visible light.

Exogenous indirect damage: This can involve photons in the UVB, UVA, and visible radiation ranges. Nitrites and nitrates are mainly activated by the UVB region. However, organic matter, the most common external sensitizers in freshwater, absorbs light in all three radiation ranges.

### 2.2. Container Material

The SODIS process requires a UV-transparent container or reactor since the solar radiation must penetrate through the material. The selection of materials for the manufacturing of SODIS containers must take into account not only optical properties but also mechanical properties, their long-term durability, and material availability [38].

#### 2.2.1. Optical Properties

SODIS mainly relies on the damage caused by solar UV radiation to microbial pathogens. However, the pathogen’s susceptibility varies with wavelength. Thus, knowing the radiation distribution at the Earth’s surface is not sufficient on its own. The wavelengths transmitted into the interior of the SODIS container is a critical factor for assessing disinfection performance.

PET bottles are the most frequently used containers for solar water disinfection. PET transmits UVA and visible light but is opaque to UVB [39], preventing the possibility of the most powerful type of direct cell damage caused by UVB radiation [38]. Alternative containers and materials that transmit UVA and UVB radiation have been successfully evaluated, including polypropylene (PP); polycarbonate (PC); polystyrene (PS) [39]; polyethylene (PE) bags [40]; polymethylmethacrylate (PMMA); [41,42]; and glass reactors (fitted with compound parabolic collectors) [43,44,45].

#### 2.2.2. Mechanical Properties

Generally, SODIS containers are used to collect, treat, and store household drinking water, which is an advantage since recontamination risk is reduced [22]. For this reason, the containers should be manufactured from robust materials that can withstand frequent handling. Sometimes, as for polyvinyl chloride (PVC), additives are added to increase the elasticity of the plastic but, in high concentrations, these can diffuse out of the plastic and into the water, posing a health risk [46]. The key mechanical properties that potential materials must guarantee are:

Resistance: Measured as tensile strength and stiffness (before failing or becoming permanently deformed) and toughness (the energy required to fracture or scratch the material).

Lightweight: Since the container may be transported every day from the house to the water source.

On the other hand, non-transportable, static SODIS systems are also used to provide safe drinking water in larger communities, such as small schools or clinics [10,42]. In this sense, good mechanical properties for the materials are not essential since the containers are less subject to falls and scratches that can decrease light transmission or cause breakages. In such circumstances, more fragile and/or heavier materials, such as glass or PMMA, can be used [47,48].

#### 2.2.3. Ageing of the Material

**Figure 2:**
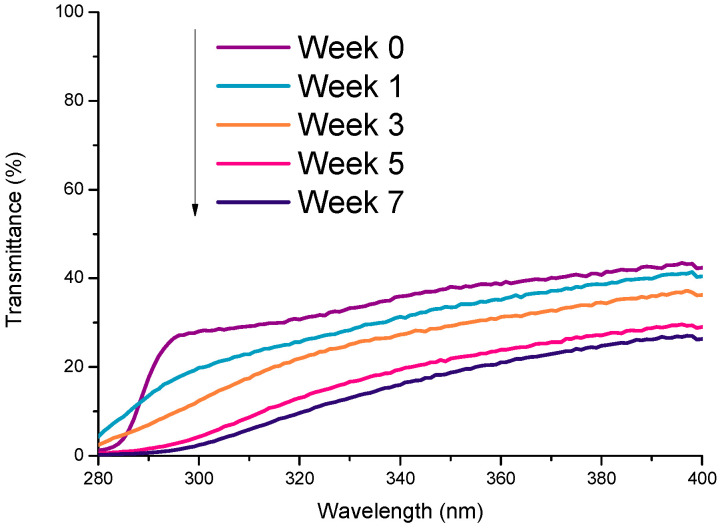
Transmittance spectra of PP samples after accelerated laboratory weathering tests in an Atlas Weather-Ometer Ci4000 according to the standard ISO 4892-2 with an intensity of 0.75 W/m^2^ at 340 nm for 7 weeks corresponding to 3.15 MJ/m^2^·nm. This dose matches with the annual dose [54]. Note: “Week” 0 is the original sample (not introduced in the Weather-Ometer).

The mechanical and optical properties of plastics can vary as a result of weathering. Figure 2 shows the results of the accelerated ageing of plastic samples to their optical transmission spectrum. The harmful effect of weather exposure on plastics is primarily attributed to photo-degradation or photo-oxidation processes by UV light and the action of oxygen [49]. Furthermore, it is well-known that temperature and humidity can speed up the degradation process [50,51].

From the perspective of photostability, plastics are grouped as follows [52]:

Poorly photostable plastics: The lifetime of these plastics is very short, usually less than one year. Some examples are PS, PVC, PP, and PE.

Moderately photostable plastics: These polymers can be used for a few years outside. Examples are PET, and PC.

Highly photostable plastics: These have an outdoor life of many years. A common example of such polymers is PMMA.

Degradation can be slowed if temperature, UV-light, and contact with oxygen and water are controlled [53]. However, this is not possible for SODIS container materials:

Avoiding unnecessary thermal exposure: The SODIS containers, by necessity, must be exposed to the sun, which will heat it. In fact, the thermal effect has been shown to accelerate the disinfection rate. Therefore, natural heating is welcomed.

Removing oxygen and water contact as much as possible: The SODIS container walls are continuously in contact with water and oxygen, from inside because they are filled with untreated water and from outside because of the atmosphere, the wind, and the humidity.

Adding UV blockers to the plastic: If UV blockers are incorporated into the plastic, the UV transmittance is reduced and, consequently, the inactivation rate is also reduced.

#### 2.2.4. Accessibility

Since solar water disinfection is designed for uptake in resource-poor environments, three more factors should be considered:

##### Affordability

SODIS is usually selected when insufficient finances are available to afford higher-price HWT. However, the selection of material must be evaluated not only in terms of efficacy (good and durable optical and mechanical properties) but also with regard to affordability. For example, PMMA is robust and highly UV transmissive, but costs twice that of PET (28.9 €/kg vs. 13.9 €/kg—data from Database 2.0 Ecoinvent [55]). Plastic ageing sometimes offsets production costs: For example, PMMA is a highly photostable plastic with many years of predicted outdoor life, whereas PET should be replaced in one or two years. However, most of the households do not have the initial investment available. If they had the funds for the more expensive material, then they would have been able to afford alternative higher-price HWT in the first place. Thus, lower-cost materials are typically used in SODIS.

##### Availability

Regions without access to safe drinking water are generally isolated, located far from the industrial centres and at the end of very long supply routes. Often, the transport cost of the material makes them too expensive, and consequently, one cannot choose the material with the best transmission or life-time characteristics. In this sense, it is always recommended to select a local container. In fact, the widespread availability of PET bottles containing bottled water or soft drinks is the main reason that PET bottles are the most frequently used SODIS containers.

##### Adoption

**Figure 3:**
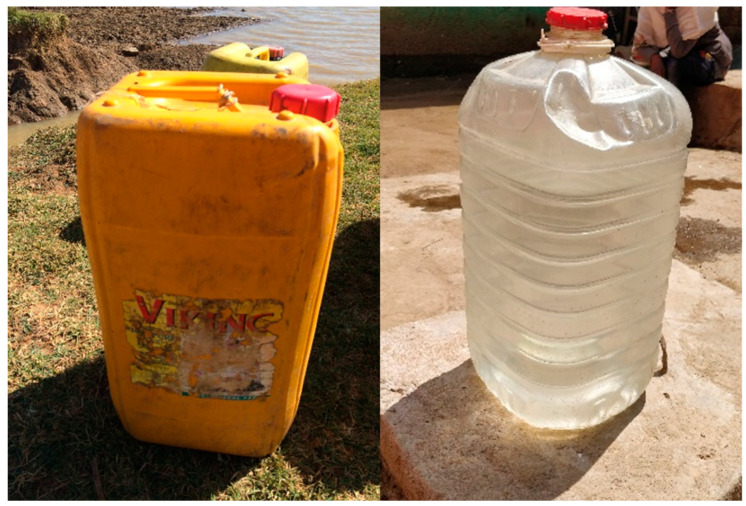
Left: Standard opaque jerrycan of 25 L used in Sub-Saharan Africa. Right: Transparent jerrycan of 25 L introduced in Ethiopian regions as SODIS container [56].

Another point to consider for optimal containers is ease of social adoption (acceptability). Several obstacles to use have been found when introducing SODIS to communities. These include scepticism due to the simplicity of the procedure, concerns about leaching of harmful substances from the plastic into the water, and the lack of promotion by bottle manufacturers [10,26]. Design of the container can improve community uptake if the design is adapted in accordance with their usual practices. For instance, in Sub-Saharan Africa, 20 L to 25 L plastic jerrycan containers are already in widespread use for the collection and transport of water. Standard jerrycans are typically made of opaque HDPE plastic. The use of transparent jerrycans that also allow the application of SODIS has been used to increase implement HWT in this region [56]. Figure 3 shows two photos of the standard opaque and transparent jerrycans.

### 2.3. Water Quality

#### 2.3.1. Chemical Composition

Freshwater can contain naturally occurring substances such as (bi)carbonates, carbohydrates, organic matters, solids, or even iron or hydrogen peroxide. Although the concentration of these substances is generally low, they can play two critical roles within the SODIS process: Either as radiation attenuators and/or as sensitizers [57].

##### Radiation Attenuators

Radiation is only slightly attenuated by pure water, which absorbs some wavelengths more than others resulting in preferential radiation concentration in the blue-visible window near the attenuation minimum [58], giving water its blue colour. However, most other substances within freshwater tend to attenuate radiation at shorter wavelengths (UV range). Water can contain suspended or dissolved natural substances. Suspended substances, such as solids, usually scatter the radiation. In contrast, dissolved substances generally absorb the radiation. Dissolved organic matter (DOM) is the main substance that absorbs radiation, especially coloured dissolved organic matter (CDOM). Scattering and absorbance increase exponentially with declining wavelengths, resulting in yellow/orange-coloured waters. Therefore, UV wavelengths tend to be strongly attenuated by naturally occurring substances [21]. As the concentration of attenuating substances is usually very low, its role can be irrelevant for small-volume containers of small dimensions (i.e., 1 L bottle). However, if the SODIS process is carried out in large-volume containers, the water quality and increased absorption-path length significantly influence the disinfection rates [57].

##### Sensitisers

Naturally occurring substances such as nitrates (NO_3_^−^), nitrites (NO_2_^−^), (bi)carbonates (HCO_3_^−^/CO_3_^−^), or CDOM can be excited by photons, which induce reactions with biomolecules through a sensitised process. In these situations, such substances are termed sensitisers. The excited chromophore can act as an oxidant or promote the formation of PPRI, such as carbonate radicals (CO_3_^•−^), the excited triplet state of CDOM (^3^CDOM*), or ROS in freshwater. Among ROS, singlet oxygen (^1^O_2_) is formed by energy transfer to dissolved oxygen, superoxide radical (O_2_^•−^) and hydrogen peroxide (H_2_O_2_) are formed by electron and proton transfer to dissolved oxygen, and hydroxyl radicals (OH^•^) are formed by Fenton reactions (hydrogen peroxide and dissolved iron), photolysis of nitrate or nitrite, or other processes involving excited chromophores [59,60,61,62,63]. Depending on the water composition, the PPRI concentrations can vary by orders of magnitude [63,64]. In addition, chromophore absorption is wavelength dependent, thus the PPRI concentrations and the resultant exogenous damage depends on the available radiation spectrum. Standard concentration ranges of PPRI in sunlit water are 10^−17^ to 10^−15^ M for OH^•^, 10^−14^ to 10^−12^ M for ^1^O_2_ and CO_3_^•−^, and 10^−12^ to 10^−10^ M for O_2_^•−^ [65]. The concentrations of PPRI for a specific water matrix and the photoreactions involved can be calculated using the APEX freeware. This software predicts the photochemical transformation kinetics of xenobiotics in surface waters as a function of kinetic variables (direct photolysis quantum yield and second-order reaction rate constants with PPRI such as OH^•^, CO_3_^•−^, ^1^O_2_ and ^3^CDOM*), water chemistry (naturally occurring substances), and the optical path length (and water depth) of sunlight in water [64,66]. The most likely reactions of PPRI with individual biomolecules have been intensively studied and are associated with electron-rich sites on biomolecules. For example, in nucleic acids, PPRI usually react with guanine [67], and in proteins, with electron-rich amino acids such as tryptophan, tyrosine, histidine, methionine, cysteine, and cystine [68,69,70,71]. However, the reactions that happen with the pathogen as a whole are unknown. In addition, PPRI attacks do not necessarily result in pathogen inactivation since they are site-specific, and many microorganisms have repair mechanisms, especially complex pathogens such as bacteria [21].

#### 2.3.2. Pathogens

The most important pathogens that cause waterborne diseases are viruses, bacteria, and protozoa:

##### Viruses

Viruses are the smallest pathogens, which are normally 0.1 µm in size. Viruses need a host cell to live, grow, and reproduce since they do not have an independent metabolism. Many viruses are host-specific, causing disease in only humans or particular animals. Rotaviruses, and hepatitis A and E viruses, are the most widespread waterborne viruses affecting humans. In 2004, rotavirus was estimated to cause over 500,000 deaths each year, with more than 85% of these occurring in low-income countries [72]. Another example is the coliphage MS2, a single-stranded RNA virus known to infect *Escherichia coli* bacteria and other Enterobacteriaceae and is commonly used as an indicator of photoinactivation due to its higher resistance in comparison to other viruses or bacteria. MS2 is also a surrogate for pathogenic enteric viruses for disinfection testing due to their similarity in morphology and survival in the environment [73,74].

**Figure 4:**
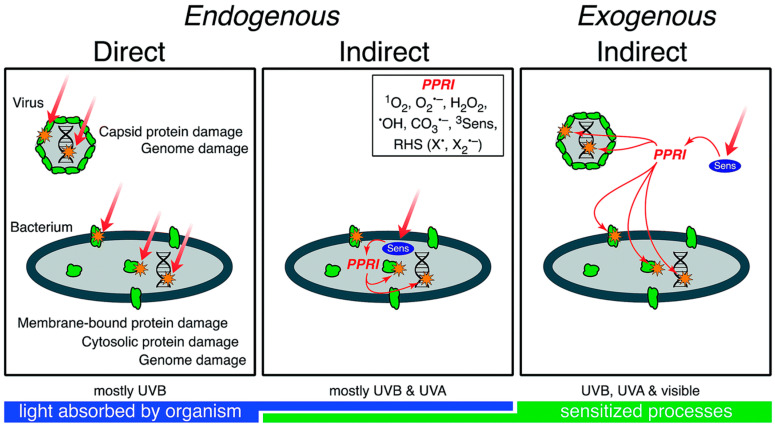
Conceptual model of sunlight inactivation mechanisms in viruses and bacteria. For direct mechanisms, the photon is absorbed by a chromophore at the site of damage (orange star). For indirect mechanisms, the photon is absorbed by a sensitiser (Sens), and damage (orange star) occurs at a different site. Green shapes represent proteins. PPRI = photo-produced reactive intermediates [21]. Reproduced from Nelson et al. Environ. Sci. Process. Impacts 2018, 20, 1089–1122, doi:10.1039/c8em00047f. Published by The Royal Society of Chemistry under a Creative Commons Attribution 3.0 Unported Licence.

Endogenous photoinactivation occurs mainly via direct damage when the genome is exposed to UVB radiation [19]. Indeed, the action spectra of photoinactivation closely mirrors the absorption spectra of RNA/DNA [75]. Due to their simple structure, consisting of a genome surrounded by a protein capsid, indirect endogenous damage is usually negligible. Regarding exogenous direct damage, external PPRI within the water matrix can inactivate viruses. Examples of harmful PPRI are singlet oxygen [71,76,77], hydroxyl radicals [37,78], carbonate radicals [37], or excited state organic matter [79]. Although all of these PPRI can inactivate viruses in isolation, their relative significance depends on the specific water characteristics and the contribution of direct inactivation (sometimes, the larger contribution of direct inactivation overshadows the exogenous inactivation) [21]. Inactivation mechanisms for viruses are summarized in Figure 4.

##### Bacteria

Bacteria are prokaryotic cells, typically of micrometre dimensions. They can live without any host since they are more complex microorganisms than viruses. Although most bacteria are harmless or even beneficial to humans, some can cause diseases such as cholera, trachoma, or salmonella. *Escherichia coli* is globally found in human and animal faeces and is a universally recognised faecal indicator. The majority of *E. coli* strains are not pathogenic; however, some strains, such as enterotoxigenic *E. coli* (ETEC), do cause disease. The Global Enteric Multicentre Study (GEMS) found ETEC to be among the top 5 pathogens most likely to produce diarrhoeal disease in children.

Bacteria can be photoinactivated by all three damage mechanisms. As bacteria have a genome, they are sensitive to direct photoinactivation by solar UVB radiation [80]. As bacteria are complex microorganisms, they contain several chromophores that produce endogenous indirect damage. In fact, even in dark conditions, bacteria can generate PPRI originating from metabolic processes. To generate energy, bacteria carry out cell respiration, involving electron transport. A small portion of free electrons interact with the oxygen in the cell interior to produce superoxide radicals and hydrogen peroxide. The latter substance can interact with internal iron by the Fenton reaction to generate hydroxyl radicals. Superoxide and hydroxyl radicals indiscriminately attack several cell targets [21]. When cells are illuminated with UVA radiation, the photosensitiser nicotinamide adenine dinucleotide in its reduced form (NADH) coenzyme, promotes superoxide formation from oxygen molecules [81]. However, bacteria have their own defence mechanisms, such as superoxide dismutase enzyme (SOD) that converts this radical to hydrogen peroxide; the catalase enzyme (CAT); and alkyl hydroperoxide reductase enzyme (Ahp) that neutralises the hydrogen peroxide [82]. These enzymes are also inactivated by UVB and UVA radiation. Furthermore, bacteria have mechanisms that repair damage caused by radical attacks and photodamage, and often recover and regrow in darkness after light exposure [83,84].

External sensitisers found in water also can damage bacteria via exogenous indirect photoinactivation under UVA radiation, specifically in Gram-positive cells: Enterococci (Gram-positive bacteria) are susceptible to indirect exogenous damage, but *E. coli* (Gram-negative bacteria) does not show noticeable inactivation [85,86]. Figure 4 sums up the inactivation mechanisms in bacteria.

##### Protozoa

Protozoa are the largest class of pathogens by size, usually about 10 to 50 µm. Protozoa are single-celled eukaryotes. Some of them form cysts in order to survive adverse conditions (such as exposure to unusual temperature, chemicals, or long periods without food or water). Many protozoa are parasites that can cause diseases such as malaria and giardiassis. In low-income countries, *Cryptosporidium parvum* protozoon is one of the top three pathogens causing diarrheal disease in children under two years old. It is responsible for 30–50% of childhood mortality [87] estimated in 455,000 annual deaths in the Sub-Saharan region [88]. Many conventional water treatments, including chlorination, are ineffective against the cysts of *C. parvum* although, the risk of infection through drinking water can be reduced by UV radiation and temperature, thus it is amenable to SODIS [89].

Solar inactivation of *C. parvum* is dominated by direct endogenous damage caused by the absorption of UVB radiation within the genome [20]. Indirect endogenous damage is negligible since the action spectrum of *C. parvum* closely resembles that of the DNA absorption [36,90]. This similarity also confirms the wavelength dependence for the photoinactivation of *C. parvum* [36,91,92]. Exogenous damage is also negligible since the presence of natural organic matter (NOM), one of the most important external sensitisers, does not cause any effect on *C. parvum* viability, most likely due to its highly resistant thick oocyst wall [90].

### 2.4. Temperature

#### 2.4.1. Inactivation Effects

Above a certain temperature, most microorganisms’ cells collapse and die. The explanation for this lack of heat-resistance is that high temperatures denature those proteins that are essential for life in microorganisms. During solar exposure, water temperature can increase significantly up to 30–50 °C. Therefore, if the pathogen contains essential proteins that are sensitive in this temperature range, it will be thermally inactivated, and these proteins will establish the thermal threshold for the pathogen. For example, cellular function in *E. coli* begins to be disrupted at 40 °C because of the melting point of the lipid membranes, whereas *T. thermophilus* bacteria proteins are unaffected up to 70 °C [93,94]. Some investigations have found that the viability of *C. parvum* protozoa progressively drops for temperatures in the range from 30 to 50 °C due to the increase in the metabolic activity and the melting point of fatty acids and hydrocarbons present in its oocyst wall [20,95,96,97,98]. Temperatures above 37 °C can induce spontaneous excystation of *C. parvum* oocysts, making their survival impossible in the absence of a host [99,100]. In the case of viruses, thermal inactivation at SODIS temperatures is usually more complicated since the virus contains fewer components. MS2 virus shows noticeable thermal inactivation above 50 °C [19,101].

In 1992, Šolić et al. [102] confirmed significant separate effects of temperature and solar radiation on the survival of faecal coliforms using analysis of variance (ANOVA) but also the dependence of the effect of one factor on the level of another. The results indicated that the effects of temperature and solar radiation are not merely additive but are synergistic. In 1998, under experimental conditions, synergistic temperature-radiation effects were found to be significant for all types of pathogens in the temperature ranges of SODIS treatment [10,103]. This synergistic effect is the result of the simultaneous action of the temperature (distributed damage caused by denaturation of components) together with the UV radiation (targeted damage triggered by absorption by chromophores). The temperature threshold and the inactivation trend vary with pathogenic species. The MS2 virus and *rotavirus* show a strong temperature dependence above 40 °C [19,104], whereas *E. coli* bacteria and *C. parvum* protozoa are susceptible above 30 °C [20,105].

#### 2.4.2. Enhancements

Various modifications to SODIS containers have been investigated in an effort to enhance temperature effects:

Painting the bottom of the container black or placing the bottles on a black surface: The non-absorbed radiation by pathogens is absorbed by the black surface, increasing water temperature by black-body radiation [106].

Using mirrors: In the SODIS process, the main aim of using mirrors is concentration of the sunlight. However, temperature also can be slightly increased as a secondary effect. Reflective surfaces or Compound Parabolic Collectors (CPC) are largely used as concentrators. The latter is usually considered to be prohibitively expensive for adoption at the household level [107,108,109]. However, high-performance, low-cost solar collectors fabricated with recycled materials, open-source hardware, and 3D-printing technologies have been developed [110].

## 3. SODIS: Kinetic Modelling

Standard SODIS guidance recommends an exposure time of 6 h on sunny days and 48 h on cloudy days. However, this is a general statement, based only on experimental results, that often overestimates the required solar exposure time. Models of the SODIS process are needed to answer the questions such as how long should the container be exposed to the sun? Reported solar disinfection rates vary over several orders of magnitude even for the same microorganisms since the kinetic models do not consider all the variables that contribute to inactivation. An accurate model should account for all the variables and parameters described in the previous section, especially for comparison of the inactivation rates from different models or to predict the required solar exposure time under field conditions.

To model the SODIS process, the most critical parameters to be considered are temperature and spectral irradiance, which are variable and unpredictable. First, precise quantitative values of these parameters must be confirmed. Then, models need to predict how both variables affect the inactivation, especially considering the potential existence of a synergistic interaction between irradiance and temperature.

**Figure 5:**
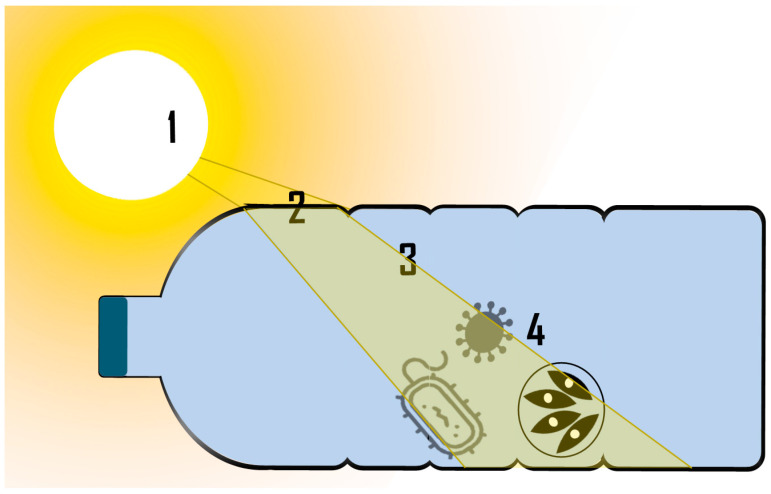
The path the radiation travels from the sun to the pathogen in the SODIS process. #1: Radiation source; #2: Crossing through the container; #3: Crossing through the water; #4 Radiation that reaches pathogens.

Summing up, the steps for modelling the SODIS process accurately are listed below:

Field values: Before modelling the kinetics, the temperature and spectral irradiance experienced by the pathogens must be determined. For the latter, the solar radiation at the container wall and the irradiance losses caused by absorption and scattering by both the container material and water matrix must be studied. Figure 5 schematically shows the pathway that the radiation follows in the SODIS process before producing damage in the microorganisms.

Modelling photoactivated processes: Identification of the photo-activated processes, the participant chromophores, the type of damage produced, and the reactions involved.

Temperature modelling: Assessment of the thermal contribution to the inactivation.

Synergy: Study of the possible synergistic effects by the joint action of irradiance and temperature.

All together: Assembly of these pieces in order to develop the entire kinetic model.

Inactivation rates can be influenced by additional factors such as abnormal pH, dissolved oxygen concentration, physiological state of microorganisms, or changes in the water matrix (for example increased concentrations of harmful substances such as hydrogen peroxide or iron). These additional processes should be considered within the kinetic model.

### 3.1. Spectral Irradiance Values

#### 3.1.1. Radiation Source

The solar radiation intensity and spectral distribution at the Earth’s surface varies with solar zenith angle (a function of latitude, time of day, and time of year) and weather conditions [43]. Solar radiation passes through the atmosphere to the Earth’s surface and is attenuated by the air mass. The path-length of the air mass depends on the relative sun position (zenith angle). The longer the path-length, the higher the radiation loss. The solar zenith angle is the angle between the sun’s rays and the normal to a plane tangent to the surface of the Earth. It varies with latitude, time of day, and time of year (season). Knowing this angle, the theoretical solar spectrum for any point on the Earth’s surface can be estimated. Changes in meteorological conditions do not imply a proportional change to the spectral radiation delivery. Visible and UVA spectral distributions are stable regardless of cloud-cover and atmospheric ozone concentrations. UVB radiation is particularly attenuated by atmospheric ozone concentration and consequently with increasing path-length. This effect varies with time of day and with season. For example, between summer and winter at mid-latitudes, UVA and visible radiation intensities vary by a factor of two, while UVB intensity varies by a factor of four [21].

Several tools can be used to estimate the theoretic sunlight spectrum as a function of zenith angle, such as the Simple Model of the Atmospheric Radiative Transfer of Sunshine (SMARTS) [111], the Tropospheric Ultraviolet and Visible Radiation Model (TUV) [33,112], or the solar calculator tool developed by Moreno-SanSegundo et al. [113]. The latter estimates both diffuse and direct irradiation from AM 0.0, introducing atmospheric extinction (atmosphere depth calculated from solar vector and elevation), absorption and scattering due to cloud coverage, and other minor contributions from temperature or humidity. Furthermore, these tools are available in software form, such as the Solar Calculator from ANSYS Fluent^®^, based on an algorithm from the National Renewable Energy Laboratory (NREL, USA) database [114]. These predictive tools usually offer an option to include a cloud-cover factor or forecast the cloud-coverage from historical data [113]. However, climate conditions such as the cloud cover are unpredictable and very influential in the spectral distribution. Thus, radiation intensity and its spectral distribution should ideally be measured in real-time during the treatment. In this sense, the use of a spectroradiometer is highly recommended in order to save the wavelength-specific irradiance over the desired range. However, the accuracy of spectroradiometer or predicting models is critical, especially in the UVB range, since kinetic rates are very sensitive to these wavelengths [21].

#### 3.1.2. Material Container

As SODIS is usually performed in a transparent container, the solar radiation that reaches the Earth’s surface is further modified when it passes through the container wall. The radiation is attenuated since containers walls absorb the radiation as a function of thickness and type of material. These variables can be easily related by the well-known Beer–Lambert law in which the absorbance is directly proportional to the path length and the extinction coefficient [115,116]. The path length is determined by the thickness of the wall, and the extinction coefficient is specific and characteristic for each material and wavelength. Note that extinction coefficients for a material are wavelength dependent. Therefore, each plastic will have an extinction coefficient spectrum, and radiation attenuation will be different for each wavelength. Some tools such as the UV Solar Calculator can be used to calculate the total radiation available and its spectral distribution inside the plastic container as a function of the thickness and type of plastic [38].

It should also be noted that the characteristic extinction coefficient spectrum of each material will change as the container ages due to weathering (Figure 2). For this reason, it is necessary to analyse the potential changes in transmission for the containers over its lifetime.

#### 3.1.3. Water Composition

**Figure 6:**
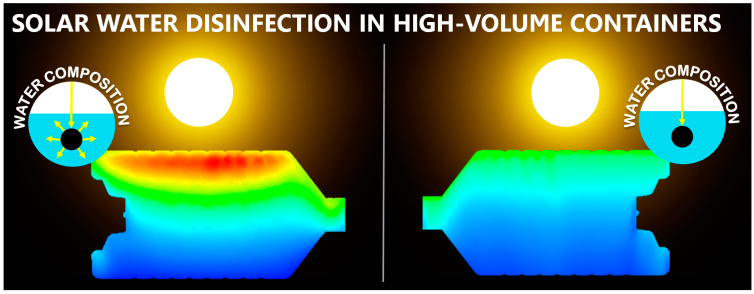
Differences in the distribution of radiation inside high-capacity containers for different optically active substances naturally occurring in water. (**Left**) substances with scattering properties. (**Right**) substances with absorption properties [57]. Warn colours (red) mean high values of irradiance, and cold colours (blue) mean low values of irradiance.

Radiation is also attenuated by water. For low volume containers (1–2 L bottles) and clear water, radiation losses related to absorption and scattering can be neglected. For natural water with low extinction, a volume-average value of the irradiance can be estimated from the attenuation provided by the Lamber–Beer law using the extinction coefficient of the water matrix. However, for large-dimension, high-volume containers, the radiation profiles within the water have to be carefully considered. The downward irradiance over a depth interval in a water column can be approximately estimated using the vertical attenuation coefficient in the downward direction [117]. This is an empirical parameter that must be measured for each particular water matrix. However, this does not account for transmission losses caused by scattering of solid particles. In this case, numerical simulation can be used to determine the irradiance distribution inside the container as a function of both absorption and scattering properties (Figure 6) (discussed in depth in Section 3.5.1).

### 3.2. Temperature

The temperature of water bodies changes depending on the solar radiation over the day and the seasons. Radiation transmission into water bodies is highly sensitive to the clarity of the water. Water bodies often experience thermal stratification in which shallower layers are brighter and warmer than the deeper layers [118]. However, due to the relatively low volume of SODIS containers, temperature gradient can be neglected.

Water temperature can be estimated by a heat balance of the water volume in the SODIS container as a function of the date using the solar altitude [119]. Several authors have used this method to estimate the water temperature even in water flowing in shade [120]. However, to achieve accurate data, experimental measurements are recommended.

### 3.3. Light Modelling

The earliest model for the inactivation of microorganisms by disinfectants derives from the Chick–Watson law [121,122]. This law states that the rate of microorganism destruction (dC/dt) is directly proportional to the number of organisms remaining at any time (C). This relation implies a uniform susceptibility of all species at a constant concentration of disinfectant—irradiance value in the case of the SODIS process—and is quantified by the kinetic constant ($k^{\prime }$). This model is based on a first-order kinetic (if the irradiance (E) that reaches the pathogen is constant) in which the slope of the linear equation is the kinetic constant expressed in time^−1^ units (k):

(1) $$\frac{dC}{dt}=-k\cdot C=-k^{\prime }\cdot E\cdot C$$

Modifications to the Chick–Watson Law have been proposed to account for deviations from simple first-order kinetics. For example, in 1972, Hom introduced an empirical generalisation to reproduce frequently observed curvilinear functions [123]. In 1978, for cases where the radiation is not constant, Chamberlin and Mitchell redefined the kinetic constant expression as the product of the kinetic constant with downward irradiance [124]. Yet another example is the series-event model proposed by Severin in 1982 that is based on the fact that microorganisms have multiple targets, all of which must be inactivated before cell death, or that a single site within the microorganisms must be hit several times before inactivation [125].

All these models are based on empirical results. Empirical models are non-selective and straightforward, so they can be adapted to other pathogens relatively easily. However, for complex systems, these models do not accurately reproduce the actual results and do not respond well to situations outside the range of the operational conditions studied (interpolation is only recommended). In contrast, mechanistic models can account for such reactions and processes. Due to their accuracy and rigour, mechanistic models can handle any operational conditions (interpolation and extrapolation) and behaviours such as synergies. However, they are more specific and more complex. A rigorous description of all involved biochemical routes is far removed from reality. Therefore, mechanistic models capture the essential steps of the global process, and are considered an optimal compromise between the fundamental description of the process and the simplicity of the model’s requirements for engineering purposes [126].

To develop kinetic models, contributions from all three types of damage (exogenous damage, direct endogenous damage, and indirect endogenous damage) should be contemplated. However, many kinetic models focus only on general endogenous damage since it is difficult to separate direct and indirect endogenous damage.

#### 3.3.1. Endogenous Damage

As the importance of the action spectrum of light in SODIS has been demonstrated previously [10,21], many authors have developed kinetic models of endogenous photoinactivation considering the irradiance distribution.

Kinetic models usually assume that all photons in a radiation range contribute to the photoinactivation in the same way. For example, Silverman et al. (2015) [127] assumed that only UVB radiation took part in the photoinactivation of MS2 virus and, therefore, only this range of radiation should account as an input parameter (E from Equation (1)) for modelling. Castro-Alférez et al. (2017) [126] studied the significant importance of UVA radiation in *E. coli* disinfection (ignoring direct damage caused by UVB radiation). They developed a mechanistic kinetic model only considering this range and defined the most ROS involved reactions that take part in the bacteria inactivation. These models fit well to the experimental results obtained with the same radiation emission spectrum. However, they do not consider radiation distribution and cannot respond appropriately to changes in the emission spectra caused when SODIS is performed with different container materials, times of year, or atmospheric conditions.

Some authors obtained the spectral action of light for photoinactivation of microorganisms using monochromatic radiation sources (via LEDs or cut-off filters). In this sense, they empirically defined a biological weighting function (P) that describes the microorganism’s sensitivity to sunlight as a function of wavelength (λ), with E(λ) being the irradiance that reaches the microorganism.

(2) $$\frac{dC}{dt}=-k\cdot C=-\int_{\lambda }P\left(\lambda \right)\cdot E\left(\lambda \right)\cdot d\lambda$$

Fisher et al. (2011) [128] defined this function for MS2 and PRD1 viruses, and Silverman et al. (2016) [129] and Lui et al. (2016) [130] also did this for different strains of *E. coli* and *enterococci* bacteria. However, these models are empirical and tell us nothing about how and why the damage occurs.

As we know, endogenous damage is produced when the internal chromophores (CHROM) are excited ($CHROM^{*}$) by the sun and, consequently, they can directly damage the microorganism (MO) or promote several harmful reactions (with PPRI as intermediates), and return to their original ground-state by emitting energy in the form of heat (infrared photons):

$$\begin{array}{ll} & CHROM^{*}+MO\to MO_{damaged}\\ CHROM+h\upsilon \to CHROM^{*} & CHROM^{*}+X\to PPRI/PPRI+MO\to MO_{damaged}\\ & CHROM^{*}\to CHROM+heat \end{array}$$

Chromophore activation is determined by its absorption spectrum and the reaction rate depends on the number of absorbed photons. Since the rate-determining step (RTD) is chromophore activation, the kinetic constant (k) can be expressed as follows:

(3) $$k=\int_{\lambda }\phi \left(\lambda \right)\cdot \varepsilon_{CHROM}\left(\lambda \right)\cdot CHROM\cdot E\left(\lambda \right)\cdot d\lambda$$

where ϕ is the quantum yield of the reaction (microorganisms damaged·per photon) or (PPRI formed per photon), $\varepsilon_{CHROM}\left(\lambda \right)$ is the specific spectral extinction coefficient of the chromophore (mL·cromophore^−1^·cm^−1^), and CHROM is the concentration of the chromophore (cromophore·mL^−1^). E(λ) is expressed in (Einstein·s^−1^·cm^−2^).

For simple microorganisms, the action spectra of photoinactivation closely mirror the absorption spectra of the RNA/DNA of many viruses and also of the absorption spectrum of the DNA of the protozoon *C. parvum* [36,75,90,128]. In these cases, the RNA/DNA is assumed to be the unique significant chromophore and, therefore, endogenous direct damage response is the only photoinactivation path. In this sense, Equation (3) can be rewritten for the endogenous photoinactivation of the microorganisms as:

(4) $$k=\phi \cdot \int_{\lambda }\varepsilon_{DNA/RNA}\left(\lambda \right)\cdot C_{DNA/RNA}\cdot E\left(\lambda \right)\cdot d\lambda$$

This method was proposed by Mattle et al. [37] to model the solar inactivation of MS2 virus from a mechanistic perspective. This kinetic model was optimised and validated with the different spectral transmittances of SODIS containers by García-Gil et al. (2020) [19]. The same procedure can be adapted to model other microorganisms inactivated via direct damage. It would only be necessary to know the genome type and size to obtain the microorganism’s absorption spectrum (the product of multiplying the DNA/RNA absorption spectrum ($\varepsilon_{DNA/RNA}$) by its concentration ($C_{DNA/RNA}$)). In this way, García-Gil et al. (2020) [20] modelled the inactivation of *C. parvum* protozoa and validated the model with different spectral transmittance from new materials of SODIS containers.

For complex microorganisms such as bacteria, many chromophores involved in the inactivation mechanisms should be considered. So far, no model has been developed to combine the action spectra with the internal reactions of the inactivation mechanisms.

In order to simplify the modelling calculations, Vione (2021) [131] published a new approach based on a monochromatic approximation to the polychromatic problem, introducing the concept of equivalent monochromatic wavelengths (EMWs). The EMW is the single wavelength that reproduces the behaviour of the poly-chromatic system, using a monochromatic (Lambert–Beer-based) equation. Following this approach, Equation (3) is transformed into:

(5) $$k=\int_{\lambda }\phi_{app}\cdot \varepsilon_{CHROM}\left(\lambda_{eq}\right)\cdot CHROM\cdot E\left(\lambda_{eq}\right)\cdot d\lambda$$

where $\phi_{app}$ is the apparent quantum yield, and $\varepsilon_{CHROM}\left(\lambda_{eq}\right)$ and $E\left(\lambda_{eq}\right)$ are the specific spectral extinction coefficients of the chromophore and the irradiance that reaches the chromophore at the equivalent wavelength ($\lambda_{eq}$), respectively. Note that $\phi_{app}$ is not exactly a quantum yield since it is the ratio between the reaction rate of a polychromatic process and the absorption of monochromatic radiation at $\lambda_{eq}$. For this reason, $\phi_{app}$ can take values up to 1. This is what happens in surface waters illuminated with the complete solar spectrum. However, for SODIS, the range of wavelengths that reach the water can vary greatly depending on the container material and may even absorb at the equivalent wavelength. In this sense, a new equivalent wavelength should be estimated for each new material.

#### 3.3.2. Exogenous Damage

Exogenous inactivation is usually modelled as a sum of the inactivation contributions generated by the PPRI detected in the water matrix. The general form to express the kinetic rates uses a second-order kinetic equation as a function of the PPRI and microorganism concentrations as follows:

(6) $$\frac{dC}{dt}=-k_{PPRRI}\cdot C\cdot \mathrm{PPRI}$$

However, determining the PPRI concentrations in the water matrix is difficult since they depend on the water composition and the spectral irradiance. Dissolved organic matter are the main substances that produce external PPRI. To define the mechanistic pathway to produce PPRI, a detailed characterisation of the water composition would be required (concentrations and action spectrum). To avoid this, the steady-state PPRI concentrations are directly measured in the water body. However, another limitation should be noted since the PPRI concentrations can vary spatially within the volume of the container due to the differences in the irradiance distribution. Freely available APEX software can be applied to address this [64,66]. The kinetic model of APEX predicts photochemical reactions and pollutant/microorganisms’ phototransformation as a function of water chemistry, for the optical path length (and water depth) of sunlight in water and its spectral distribution. The model applies Equation (5) for each water substance to calculate the PPRI concentration and later uses Equation (6) to calculate the disinfection rate for the exogenous damage. However, key input data on pollutant/microorganism photoreactivity parameters such as the direct photolysis quantum yield and the second-order reaction rate constants with OH^•^, CO_3_^•−^, ^1^O_2_, and ^3^CDOM* are required. Second-order rate constants have been reported in the literature for different viruses (MS2, PhiiX174, HadV, and rotavirus) and PPRI (singlet oxygen, hydroxyl radical, carbonate radicals, and excited dissolved organic matter) [37,76,79,127]. However, in some cases, different values of the kinetic constant are reported for the same reaction. For example, the second-order kinetic constant for MS2 inactivation with the singlet oxygen PPRI was reported as 3.1·10^9^ M^−1^s^−1^ by Mattle et al. [37] and as 3.8·10^8^ M^−1^s^−1^ by Silverman et al. [127]. These discrepancies can be caused by overstated assumptions or differences between the sensitiser–virus association that depends on the water matrix [21]. In addition, as PPRI promotion depends on the excitation of the chromophores within the body of water, the water composition and the spectral irradiance can play important roles. For these reasons, it is recommended to obtain the specific kinetic constants for each specific water.

### 3.4. Temperature Modelling

Other modifications derived from the Chick–Watson Law have been used to model thermal inactivation. In 1978, Mancini [132] defined the kinetic constant by an exponential function depending on the temperature. Later, this thermal inactivation model was adopted by Peng et al. [96] for the modelling of the *C. parvum* protozoa inactivation as well as by McGuigan et al. [103] for the modelling of the *E. coli* bacteria die-off. In fact, this publication was the first kinetics approach considering the radiation-temperature synergistic effect. They reported a synergy parameter that multiplied the sum of the light and temperature kinetic constants. Values of this parameter larger than 1 indicate that synergy happens.

All the previous approaches are very close to the well-known Arrhenius equation, which has been widely used for modelling temperature dependence of reaction rates (complex as well as elementary reactions) expressing the thermal kinetic constant ($k_{T}$) as a function of temperature (T) as follows:

(7) $$k_{T}=k_{o}\cdot \exp\left(-\frac{Ea}{R\cdot T}\right)$$

where R is the universal gas constant, Ea is the activation energy, and $k_{o}$ is the pre-exponential factor. This equation is seen as an empirical relation since $k_{o}$ and Ea are temperature-independent constants experimentally determined for each reaction. Despite this consideration, Arrhenius provided a physical explanation for the equation since the activation energy concept indicates the minimum amount of energy acquired by substances to react. This term justifies the exponential nature of the relationship and can be calculated from statistical methods.

For reactions in which the relation between the kinetic rate and temperature are larger than exponential, the variant Modified Arrhenius equation (Equation (8)) can be used:

(8) $$k_{T}={k_{o}}^{\prime }\cdot T^{n}\cdot \exp\left(-\frac{Ea}{R\cdot T}\right)$$

where the pre-exponential factor is proportional to $T^{n}$ where T is the temperature and n a constant. If *n* takes the value 1.0, this variant becomes in the original Arrhenius Equation.

This Arrhenius-like equation can be rewritten by introducing a threshold temperature ($T_{0}$) as follows:

(9) $$k_{T}=k_{o}\cdot \exp\left(-\frac{Ea}{R}\cdot \left(\frac{1}{T}-\frac{1}{T_{0}}\right)\right)$$

As Peleg et al. (2012) [133] demonstrated, the threshold temperature can be suppressed, which involves a different value of $k_{0}$ for the same value of k. However, the temperature threshold can be kept as a conceptual threshold to account for the temperature above which the thermal effect is observed.

The Arrhenius equation approach was used by Castro-Alférez et al. (2017) [105] to include the inactivation of *E. coli* bacteria in the dark as well as the UV&T synergistic effect, including this temperature dependence in the photoinactivation kinetic constant of their mechanistic model. However, this kinetic model does not consider the action spectral (the reactions are described like Equation (1)). The integration of both action spectral and synergistic effect can be possible if the reactions are described as in Equation (3) and the quantum yield is expressed as temperature dependent. This technique was used by García-Gil et al. (2020) [19] to model the MS2 virus inactivation and by García-Gil et al. (2020) [20] to model the *C. parvum* protozoa inactivation.

### 3.5. Comprehensive Kinetic Models

A comprehensive kinetic model must consider all significant factors affecting the microorganism inactivation. Once all the significant photo-activated processes and thermal effects are identified and kinetically described following the above-mentioned approaches, the microorganisms’ inactivation balance can be solved. For that, the microorganism die-off depends on all the reactions related to endogenous damage (endogenous chromophores), synergistic effects, exogenous damage (external PPRI), and dark inactivation (usually thermal inactivation):

(10) $$\frac{dC}{dt}=-\left(k_{endo}+k_{exo}+k_{dark}\right)\cdot C$$

where:

$$\begin{array}{l} k_{endo}=\sum \int_{\lambda }\phi \left(\lambda ,T\right)\cdot \varepsilon_{CHROM}\left(\lambda \right)\cdot CHROM\cdot E\left(\lambda \right)\cdot d\lambda \\ k_{exo}=\sum k_{PPRRI}\cdot \mathrm{PPRI}\\ k_{dark}=k_{T} \end{array}$$

However, some reaction rates can depend on other non-constant substances. Thus, the mass balance of these substances must also be taken into account.

If irradiance is homogenous inside the SODIS container, Equation (10) can be solved because E(λ) is constant. If irradiance is not homogenous, but the water is well-mixed, Equation (10) can be solved using a unique value of E(λ) that represents the average incident radiation in the total volume. This value can be obtained from actinometry or using Computational Fluid Dynamics (CFD) techniques based on numerical simulations. If the irradiance is not homogeneous and the reactor is not well-mixed, Equation (10) must be simultaneously solved in each differential volume, and this is only possible using CFD techniques.

#### 3.5.1. Computational Fluid Dynamics (CFD)

Solving numerical equations in silico is usually accomplished using Computational Fluid Dynamics (CFD). This technique has been shown to be a very promising tool in the design, optimisation, and up-scaling of fluid systems since it saves time, cost, and effort. It is based on dividing the space into numerous discrete cells and solving the equations in each cell for all the phenomena involved. Its main advantage in photoactivated processes resides in the possibility of coupling rigorous calculations of the radiative transport equation (RTE), with hydrodynamics, radiation transfer, mass transport, and chemical reaction rate within the reactor.

The RTE is an integro-differential equation that describes the journey of photonic rays through the volume with their corresponding energy-loss due to absorption and out-scattering, and energy-gain due to in-scattering from other directions. In the case of the SODIS process, the water matrix can be considered as a homogeneous body, and the emission of radiation can be neglected due to the operational temperatures. In this sense, the RTE takes the following form [134,135]:

(11) $$\frac{dI_{\lambda ,\underline{\Omega }}}{ds}=-\kappa_{\lambda }I_{\lambda ,\underline{\Omega }}-\sigma_{\lambda }I_{\lambda ,\underline{\Omega }}+\frac{\sigma_{\lambda }}{4\pi }\int_{\Omega^{\prime }=4\pi }p\left({\underline{\Omega }}^{\prime }\to \underline{\Omega }\right)I_{\lambda ,{\underline{\Omega }}^{\prime }}d\Omega^{\prime }$$

where $I_{\lambda ,\underline{\Omega }}$ is the intensity of photons with wavelength λ propagated along direction Ω, s is the differential space, $\kappa_{\lambda }$ is the volumetric absorption coefficient, $\sigma_{\lambda }$ is the volumetric scattering coefficient, and $p\left({\underline{\Omega }}^{\prime }\to \underline{\Omega }\right)$ is the phase function that describes the directional distribution of scattered radiation. The solution of this equation allows the evaluation of the radiation field at any point (differential space) inside the reactor volume.

Solving the RTE can be accomplished using a number of different approaches. The Discrete Ordinate Method (DOM) solves the radiation field at any point inside the geometry for a finite number of discrete solid angles, with each one associated with a direction vector. This method is the most versatile and rigorous, since it allows consideration of the wavelength the emission, absorption, and scattering properties of surfaces and volumes. It is also valid for the whole range of optical thicknesses and the solution or radiation transport through semi-transparent walls. When the DOM is used, the spatial discretisation of the computational region is taken directly from the mesh grid topology. However, the directional discretisation for the RTE is explicitly specified using an angular discretisation of the sphere octant in *NθxNϕ* solid angles, also called control angles, conforming to the directions in which the RTE is solved. The selection of the angular discretisation and the meshing must be carefully studied to guarantee independent results from the complexity of angular and space discretisation. However, the higher the number of divisions, the higher the computational costs. Thus, a balance must be found.

Once the incident radiation distribution is known, the average can be obtained by integrating in the volume or surface of interest or can be accounted for in each cell to solve differentially other phenomena such as the kinetic model. Some CFD software can be used to solve the numerical equations of the radiation field. For example, OpenFOAM is a free, open-source CFD software with great capabilities, where users can customise the solutions for a specific problem. Recently, the Discrete Ordinate Method (DOM) was developed and implemented within this simulation framework [136]. Commercial CFD software such as ANSYS Fluent or COMSOL also include the solution of the RTE. García-Gil et al. (2020) [57] used Ansys Fluent software to determine that bicarbonates, soluble carbohydrates, humic acids, and solids play the main role of radiation attenuators in the SODIS process for large-volume containers. In addition, they developed a predictive model that estimates the required solar exposure time based on the average radiation intensity and its uniformity within the container, depending on naturally occurring substances in the water (Figure 6).

## 4. Conclusions

Nowadays, three main challenges have been identified that slow down the adoption of the standard SODIS process despite it being one of the most accessible and cheap HWT:

(1) Low batch volume of bottles.
(2) Limited effectiveness of PET bottles against viruses and protozoa.
(3) Overestimation of the recommended exposure time.

New enhancements are being studied to overcome these limitations. Using a large number of 2 L bottles to meet the daily requirements for safe drinking water consumption increases the risk of recontamination. In this sense, scale-up from one 2 L bottle to a large-capacity container (20–25 L) is a possible solution to reduce the number of containers. However, increasing the volume of the SODIS containers must be carefully addressed to ensure that the effect of water characteristics on the radiation distribution (absorption and scattering) is considered to conduct an in-depth evaluation of the radiation reaching pathogens. Accurate estimation of the radiation distribution could require the use of CFD software to solve complex numerical equations saving costs, efforts, and time but also requiring specific training and computational costs.

Regarding the limitations of PET-based SODIS against viruses and protozoa, some researchers have focused on the study of other potential materials for manufacturing SODIS reactors to ensure disinfection or to reduce the required solar exposure time. However, the selection of new materials must also consider their optical and mechanical properties, durability, and availability. Note that the SODIS process is one of the cheapest HWT. Thus, the selection of new materials cannot increase the costs, or the durability of the container must compensate for any increase in cost. However, often the households do not have the initial investment required, so they use the lowest-cost materials.

The fact that the recommended exposure time is overestimated originates from discrepancies of reported solar disinfection rates, sometimes even for the same microorganisms. The reason that this happens is that most kinetic models do not account for all the variables that contribute towards inactivation: Irradiance, spectral distribution, temperature, container material, water composition, and pathogenic species, all drastically modify inactivation efficacy. An accurate model must consider all the variables discussed in the previous section, especially to compare inactivation rates from different models or predict the required solar exposure time under variable field conditions. Closer consideration of new materials and size of the SODIS reactors, as well as the development of more accurate kinetic models, will make the SODIS process faster and safer and will undoubtedly contribute towards more widespread adoption and uptake.

## Data Availability

Supporting dataset available at https://doi.org/10.5281/zenodo.4899573.

## References

[1] United Nations (UN) (2010). A/RES/64/292.

[2] United Nations (UN) (2015). The 2030 AGENDA for Sustainable Development.

[3] United Nations (UN) (2018). Sustainable Development Goal 6. Synthesis Report on Water and Sanitation 2018.

[4] UNU-INWEH/UNESCAP (2013). Water Security & the Global Water Agenda: A UN-Water Analytical Brief.

[5] FAO (2017). The Future of Food and Agriculture: Trends and Challenges.

[6] IPCC (2018). Summary for Policymakers. Global Warming of 1.5 °C.

[7] Wada Y., van Beek L.P.H., Wanders N. (2014). Sustainability of global water use: Past reconstruction and future projections Related content Human water consumption intensifies hydrological drought worldwide. Environ. Res. Lett..

[8] UNESCO/UN-Water (2020). The United Nations World Water Development Report 2020: Water and Climate Change.

[9] Institute for Health Metrics and Evaluation Global Burden of Disease (GBD) Compare. https://www.thelancet.com/gbd/gbd-compare-visualisation.

[10] McGuigan K.G., Conroy R.M., Mosler H.-J., du Preez M., Ubomba-Jaswa E., Fernandez-Ibañez P. (2012). Solar water disinfection (SODIS): A review from bench-top to roof-top. J. Hazard. Mater..

[11] Gadgil A. (1998). Drinking water in developing countries. Annu. Rev. Energy Environ..

[12] Pichel N., Vivar M., Fuentes M. (2019). The problem of drinking water access: A review of disinfection technologies with an emphasis on solar treatment methods. Chemosphere.

[13] World Health Organization (WHO) Boil Water. https://www.who.int/water_sanitation_health/dwq/Boiling_water_01_15.pdf.

[14] Clasen T., McLaughlin C., Nayaar N., Boisson S., Gupta R., Desai D., Shah N. (2008). Microbiological effectiveness and cost of disinfecting water by boiling in semi-urban India. Am. J. Trop. Med. Hyg..

[15] Gilman R.H., Skillicorn P. (1985). Boiling of drinking-water: Can a fuel-scarce community afford it?. Bull. World Health Organ..

[16] Clasen T., Haller L., Walker D., Bartram J., Cairncross S. (2007). Cost-effectiveness of water quality interventions for preventing diarrhoeal disease in developing countries. J. Water Health.

[17] World Health Organization (WHO) (2017). Regional Office for South.-East. Asia Principles and Practices of Drinking-Water Chlorination: A Guide to Strengthening Chlorination Practices in Small-to Medium Sized Water Supplies.

[18] World Health Organization (WHO) (2002). Managing Water in the Home: Accelerated Health Gains from Improved Water Supply.

[19] García-Gil Á., Martinez A., Polo-López M.I., Marugán J. (2020). Kinetic modeling of the synergistic thermal and spectral actions on the inactivation of viruses in water by sunlight. Water Res..

[20] García-Gil Á., Abeledo-Lameiro M.J., Gómez-Couso H., Marugán J. (2020). Kinetic modeling of the synergistic thermal and spectral actions on the inactivation of *Cryptosporidium parvum* in water by sunlight. Water Res..

[21] Nelson K.L., Boehm A.B., Davies-Colley R.J., Dodd M.C., Kohn T., Linden K.G., Liu Y., Maraccini P.A., McNeill K., Mitch W.A. (2018). Sunlight-mediated inactivation of health-relevant microorganisms in water: A review of mechanisms and modeling approaches. Environ. Sci. Process. Impacts.

[22] Rufener S., Mäusezahl D., Mosler H.-J., Weingartner R. (2010). Quality of drinking-water at source and point-of-consumption-drinking cup as a high potential recontamination risk: A field study in Bolivia. J. Health Popul. Nutr..

[23] Luzi S., Tobler M., Suter F., Meierhofer R. (2016). SODIS Manual: Guidance on Solar Water Disinfection.

[24] Wegelin M., Canonica S., Alder A.C., Marazuela D., Suter M.J.-F., Bucheli T.D., Haefliger O.P., Zenobi R., McGuigan K.G., Kelly M.T. (2001). Does sunlight change the material and content of polyethylene terephthalate (PET) bottles?. Res. Technol..

[25] Ubomba-Jaswa E., Fernández-Ibáñez P., McGuigan K.G. (2010). A preliminary Ames fluctuation assay assessment of the genotoxicity of drinking water that has been solar disinfected in polyethylene terephthalate (PET) bottles. J. Water Health.

[26] Ozores Diez P., Giannakis S., Rodríguez-Chueca J., Wang D., Quilty B., Devery R., McGuigan K., Pulgarin C. (2020). Enhancing solar disinfection (SODIS) with the photo-Fenton or the Fe^2+^/peroxymonosulfate-activation process in large-scale plastic bottles leads to toxicologically safe drinking water. Water Res..

[27] World Health Organization (WHO) (2005). Emergency Treatment of Drinking Water at Point-of-Use. WHO-Technical Notes for Emergencies No. 5.

[28] World Health Organization (WHO) (2011). Evaluating Household Water Treatment Options: Health-Based Targets and Microbiological Performance Specifications.

[29] Center for Desease Control and Prevention (CDC) Household Water Treatment. Solar Disinfection. https://www.cdc.gov/safewater/pdf/solar2011final.pdf.

[30] Conroy R.M., Meegan M.E., Joyce T., McGuigan K., Barnes J. (2001). Solar disinfection of drinking water protects against cholera in children under 6 years of age. Arch. Dis. Child..

[31] Graf J., Togouet S.Z., Kemka N., Niyitegeka D., Meierhofer R., Pieboji J.G. (2010). Health gains from solar water disinfection (SODIS): Evaluation of a water quality intervention in Yaoundé, Cameroon. J. Water Health.

[32] Rose A., Roy S., Abraham V., Holmgren G., George K., Balraj V., Abraham S., Muliyil J., Joseph A., Kang G. (2006). Solar disinfection of water for diarrhoeal prevention in southern India. Arch. Dis. Child..

[33] Kohn T., Mattle M.J., Minella M., Vione D. (2016). A modeling approach to estimate the solar disinfection of viral indicator organisms in waste stabilization ponds and surface waters. Water Res..

[34] Parker K.M., Mitch W.A. (2016). Halogen radicals contribute to photooxidation in coastal and estuarine waters. Proc. Natl. Acad. Sci. USA.

[35] Silverman A.I., Tay N., Machairas N. (2019). Comparison of biological weighting functions used to model endogenous sunlight inactivation rates of MS2 coliphage. Water Res..

[36] Busse M.M., Becker M., Applegate B.M., Camp J.W., Blatchley E.R. (2019). Responses of *Salmonella typhimurium* LT2, *Vibrio harveyi*, and *Cryptosporidium parvum* to UVB and UVA radiation. Chem. Eng. J..

[37] Mattle M.J., Vione D., Kohn T. (2015). Conceptual model and experimental framework to determine the contributions of direct and indirect photoreactions to the solar disinfection of MS2, phiX174, and adenovirus. Environ. Sci. Technol..

[38] García-Gil Á., Pablos C., García-Muñoz R.A., McGuigan K.G., Marugán J. (2020). Material selection and prediction of solar irradiance in plastic devices for application of solar water disinfection (SODIS) to inactivate viruses, bacteria and protozoa. Sci. Total Environ..

[39] Fisher M.B., Iriarte M., Nelson K.L. (2012). Solar water disinfection (SODIS) of *Escherichia coli, Enterococcus* spp., and MS2 coliphage: Effects of additives and alternative container materials. Water Res..

[40] Lawrie K., Mills A., Figueredo-Fernández M., Gutiérrez-Alfaro S., Manzano M., Saladin M. (2015). UV dosimetry for solar water disinfection (SODIS) carried out in different plastic bottles and bags. Sens. Actuators B Chem..

[41] Ubomba-Jaswa E., Fernández-Ibáñez P., Navntoft C., Polo-López M.I., McGuigan K.G. (2010). Investigating the microbial inactivation efficiency of a 25 L batch solar disinfection (SODIS) reactor enhanced with a compound parabolic collector (CPC) for household use. J. Chem. Technol. Biotechnol..

[42] Reyneke B., Ndlovu T., Vincent M.B., Martínez-García A., Polo-López M.I., Fernández-Ibáñez P., Ferrero G., Khan S., McGuigan K.G., Khan W. (2020). Validation of large-volume batch solar reactors for the treatment of rainwater in field trials in sub-Saharan Africa. Sci. Total Environ..

[43] García-Gil Á., Casado C., Pablos C., Marugán J. (2019). Novel procedure for the numerical simulation of solar water disinfection processes in flow reactors. Chem. Eng. J..

[44] Mac Mahon J., Gill L.W. (2018). Sustainability of novel water treatment technologies in developing countries: Lessons learned from research trials on a pilot continuous flow solar water disinfection system in rural Kenya. Dev. Eng..

[45] Kalt P., Birzer C., Evans H., Liew A., Padovan M., Watchman M. (2014). A solar disinfection water treatment system for remote communities. Procedia Eng..

[46] Wegelin M., Sommer B. (1998). Solar water disinfection (SODIS)-destined for worldwide use?. Waterlines.

[47] Fagan H.G., Linnane K.S., McGuigan K.G., Rugamayo A. (2015). Water is Life—Progress to Secure Water Provision in Rural Uganda.

[48] Martínez-García A., Vincent M., Rubiolo V., Domingos M., Canela M.C., Oller I., Fernández-Ibáñez P., Polo-López M.I. (2020). Assessment of a pilot solar V-trough reactor for solar water disinfection. Chem. Eng. J..

[49] Gillen K.T., Celina M. (2017). Predicting polymer degradation and mechanical property changes for bombined radiation-thermal aging environments. Rubber Chem. Technol..

[50] Martin J.R., Gardner R.J. (1981). Effect of long term humid aging on plastics. Polym. Eng. Sci..

[51] White J.R. (2006). Polymer ageing: Physics, chemistry or engineering? Time to reflect. Comptes Rendus Chim..

[52] Rånby B. (1993). Basic reactions in the photodegradation of some important polymers. J. Macromol. Sci. Part A Pure Appl. Chem..

[53] Kircher K. (1987). Chemical Reactions in Plastics Processing.

[54] Kuvshinnikova O., Boven G., Pickett J.E. (2019). Weathering of aromatic engineering thermoplastics: Comparison of outdoor and xenon arc exposures. Polym. Degrad. Stab..

[55] Frischknecht R., Jungbluth N., Althaus H.-J., Doka G., Dones R., Heck T., Hellweg S., Hischier R., Nemecek T., Rebitzer G. (2005). The ecoinvent database: Overview and methodological framework. Int. J. Life Cycle Assess..

[56] WaterSPOUTT Project. www.waterspoutt.eu.

[57] García-Gil Á., Valverde R., García-Muñoz R.A., McGuigan K.G., Marugán J. (2020). Solar Water Disinfection in high-volume containers: Are naturally occurring substances attenuating factors of radiation?. Chem. Eng. J..

[58] Gall M.P., Davies-Colley R.J., Merrilees R.A. (2013). Exceptional visual clarity and optical purity in a sub-alpine lake. Limnol. Oceanogr..

[59] Vione D., Minella M., Maurino V., Minero C. (2014). Indirect photochemistry in sunlit surface waters: Photoinduced production of reactive transient species. Chemistry.

[60] McNeill K., Canonica S. (2016). Triplet state dissolved organic matter in aquatic photochemistry: Reaction mechanisms, substrate scope, and photophysical properties. Environ. Sci. Process. Impacts.

[61] Zafiriou O.C. (1974). Sources and reactions of OH and daughter radicals in seawater. J. Geophys. Res..

[62] Hoigné J., Faust B.C., Haag W.R., Scully F.E., Zepp R.G. (1988). Aquatic humic substances as sources and sinks of photochemically produced transient reactants. Aquatic Humic Substances.

[63] Foote C.S., Selverstone Valentine J., Arthur G. (1995). Active Oxygen in Chemistry.

[64] Bodrato M., Vione D. (2014). APEX (Aqueous Photochemistry of Environmentally occurring Xenobiotics): A free software tool to predict the kinetics of photochemical processes in surface waters. Environ. Sci. Process. Impacts.

[65] Schwarzenbach R.P., Gschwend P.M., Imboden D.M. (1993). Environmental Organic Chemistry.

[66] Vione D. (2020). A critical view of the application of the APEX software (Aqueous Photochemistry of Environmentally-Occurring Xenobiotics) topredict photoreaction kinetics in surface freshwaters. Molecules.

[67] Smit K.C., Smit K.C. (1989). The Science of Photobiology.

[68] Davies M.J. (2003). Singlet oxygen-mediated damage to proteins and its consequences. Biochem. Biophys. Res. Commun..

[69] Boreen A.L., Edhlund B.L., Cotner J.B., McNeill K. (2008). Indirect photodegradation of dissolved free amino acids: The contribution of singlet oxygen and the differential reactivity of DOM from various sources. Environ. Sci. Technol..

[70] Lundeen R.A., Janssen E.M.-L., Chu C., Mcneill K. (2014). Environmental Photochemistry of Amino Acids, Peptides and Proteins. Chimia.

[71] Michaeli A., Feitelson J. (1994). Reactivity of singlet oxygen toward amino acids and peptides. Photochem. Photobiol..

[72] Parashar U.D., Burton A., Lanata C., Boschi-Pinto C., Shibuya K., Steele D., Birmingham M., Glass R.I. (2009). Global mortality associated with rotavirus disease among children in 2004. J. Infect. Dis..

[73] Love D.C., Silverman A., Nelson K.L. (2010). Human virus and bacteriophage inactivation in clear water by simulated sunlight compared to bacteriophage inactivation at a Southern California beach. Environ. Sci. Technol..

[74] Theitler D.J., Nasser A., Gerchman Y., Kribus A., Mamane H. (2012). Synergistic effect of heat and solar UV on DNA damage and water disinfection of *E. Coli* and bacteriophage MS2. J. Water Health.

[75] Lytle C.D., Sagripanti J.-L. (2005). Predicted inactivation of viruses of relevance to biodefense by solar radiation. J. Virol..

[76] Kohn T., Nelson K.L. (2007). Sunlight-mediated inactivation of MS2 coliphage via exogenous singlet oxygen produced by sensitizers in natural waters. Environ. Sci. Technol..

[77] Kohn T., Grandbois M., Mcneill K., Nelson K.L. (2007). Association with natural organic matter enhances the sunlight-mediated inactivation of MS2 coliphage by singlet oxygen. Environ. Sci. Technol..

[78] Romero-Maraccini O.C., Sadik N.J., Rosado-Lausell S.L., Pugh C.R., Niu X.-Z., Croué J.-P., Nguyen T.H. (2013). Sunlight-induced inactivation of human Wa and porcine OSU rotaviruses in the presence of exogenous photosensitizers. Environ. Sci. Technol..

[79] Rosado-Lausell S.L., Wang H., Gutiérrez L., Romero-Maraccini O.C., Niu X.Z., Gin K.Y.H., Croué J.P., Nguyen T.H. (2013). Roles of singlet oxygen and triplet excited state of dissolved organic matter formed by different organic matters in bacteriophage MS2 inactivation. Water Res..

[80] Jagger J. (1985). Solar-UV Actions on Living Cells.

[81] Chen S., Schopfer P. (1999). Hydroxyl-radical production in physiological reactions. Eur. J. Biochem..

[82] Seaver L.C., Imlay J.A. (2001). Alkyl hydroperoxide reductase is the primary scavenger of endogenous hydrogen peroxide in *Escherichia coli*. J. Bacteriol..

[83] Giannakis S., Darakas E., Escalas-Cañellas A., Pulgarin C. (2015). Solar disinfection modeling and post-irradiation response of Escherichia coli in wastewater. Chem. Eng. J..

[84] Sinha R.P., Häder D.-P. (2002). UV-induced DNA damage and repair: A review. Photochem. Photobiol. Sci..

[85] Maraccini P.A., Wenk J., Boehm A.B. (2016). Exogenous indirect photoinactivation of bacterial pathogens and indicators in water with natural and synthetic photosensitizers in simulated sunlight with reduced UVB. J. Appl. Microbiol..

[86] Maraccini P.A., Wenk J., Boehm A.B. (2016). Photoinactivation of eight health-relevant bacterial species: Determining the importance of the exogenous indirect mechanism. Environ. Sci. Technol..

[87] Kotloff K.L., Nataro J.P., Blackwelder W.C., Nasrin D., Farag T.H., Panchalingam S., Wu Y., Sow S.O., Sur D., Breiman R.F. (2013). Burden and aetiology of diarrhoeal disease in infants and young children in developing countries (the Global Enteric Multicenter Study, GEMS): A prospective, case-control study. Lancet.

[88] Sow S.O., Muhsen K., Nasrin D., Blackwelder W.C., Wu Y., Farag T.H., Panchalingam S., Sur D., Zaidi A.K.M., Faruque A.S.G. (2016). The burden of *Cryptosporidium* diarrheal disease among children <24 months of age in moderate/high mortality regions of Sub-Saharan Africa and South Asia, utilizing data from the Global Enteric Multicenter Study (GEMS). PLoS Negl. Trop. Dis..

[89] Gómez-Couso H., Fontán-Sainz M., Navntoft C., Fernández-Ibáñez P., Ares-Mazás E. (2012). Comparison of different solar reactors for household disinfection of drinking water in developing countries: Evaluation of their efficacy in relation to the waterborne enteropathogen *Cryptosporidium parvum*. Trans. R. Soc. Trop. Med. Hyg..

[90] Liu Y., Dong S., Kuhlenschmidt M.S., Kuhlenschmidt T.B., Drnevich J., Nguyen T.H. (2015). Inactivation mechanisms of *Cryptosporidium parvum* oocysts by solar ultraviolet irradiation. Environ. Sci. Water Res. Technol..

[91] Linden K.G., Shin G., Sobsey M.D. (2001). Comparative effectiveness of UV wavelengths for the inactivation of *Cryptosporidium parvum* oocysts in water. Water Sci. Technol..

[92] Beck S.E., Wright H.B., Hargy T.M., Larason T.C., Linden K.G. (2015). Action spectra for validation of pathogen disinfection in medium-pressure ultraviolet (UV) systems. Water Res..

[93] Leuenberger P., Ganscha S., Kahraman A., Cappelletti V., Boersema P.J., von Mering C., Claassen M., Picotti P. (2017). Cell-wide analysis of protein thermal unfolding reveals determinants of thermostability. Science.

[94] Mackey B.M., Miles C.A., Parsons S.E., Seymour D.A. (1991). Thermal denaturation of whole cells and cell components of *Escherichia coli* examined by differential scanning calorimetry. J. Gen. Microbiol..

[95] Fayer R., Nerad T. (1996). Effects of low temperatures on viability of *Cryptosporidium parvum* oocysts. Appl. Environ. Microbiol..

[96] Peng X., Murphy T., Holden N.M. (2008). Evaluation of the effect of temperature on the die-off rate for *Cryptosporidium parvum* oocysts in water, soils, and feces. Appl. Environ. Microbiol..

[97] Jenkins M.B., Eaglesham B.S., Anthony L.C., Kachlany S.C., Bowman D.D., Ghiorse W.C. (2010). Significance of wall structure, macromolecular composition, and surface polymers to the survival and transport of *Cryptosporidium parvum* Oocysts. Appl. Environ. Microbiol..

[98] King B.J., Keegan A.R., Monis P.T., Saint C.P. (2005). Environmental temperature controls Cryptosporidium oocyst metabolic rate and associated retention of infectivity. Appl. Environ. Microbiol..

[99] Gómez-Couso H., Fontán-Sainz M., Fernández-Alonso J., Ares-Mazás E. (2009). Excystation of *Cryptosporidium parvum* at temperatures that are reached during solar water disinfection. Parasitology.

[100] Smith H.V., Nichols R.A.B., Grimason A.M. (2005). *Cryptosporidium* excystation and invasion: Getting to the guts of the matter. Trends Parasitol..

[101] Seo K., Lee J.E.U.N., Lim M.I.Y., Ko G. (2012). Effect of temperature, pH, and NaCl on the inactivation kinetics of murine norovirus. J. Food Prot..

[102] Šolić M., Krstulović N. (1992). Separate and combined effects of solar radiation, temperature, salinity, and pH on the survival of faecal coliforms in seawater. Mar. Pollut. Bull..

[103] McGuigan K.G., Joyce T.M., Conroy R.M., Gillespie J.B., Elmore-Meegan M. (1998). Solar disinfection of drinking water contained in transparent plastic bottles: Characterizing the bacterial inactivation process. J. Appl. Microbiol..

[104] Romero O.C., Straub A.P., Kohn T., Nguyen T.H. (2011). Role of temperature and Suwannee River Natural Organic Matter on inactivation kinetics of rotavirus and bacteriophage MS2 by solar irradiation. Environ. Sci. Technol..

[105] Castro-Alférez M., Polo-López M.I., Marugán J., Fernández-Ibáñez P. (2017). Mechanistic modeling of UV and mild-heat synergistic effect on solar water disinfection. Chem. Eng. J..

[106] Mani S.K., Kanjur R., Bright Singh I.S., Reed R.H. (2006). Comparative effectiveness of solar disinfection using small-scale batch reactors with reflective, absorptive and transmissive rear surfaces. Water Res..

[107] Casado C., García-Gil Á., van Grieken R., Marugán J. (2019). Critical role of the light spectrum on the simulation of solar photocatalytic reactors. Appl. Catal. B Environ..

[108] Kehoe S.C., Joyce T.M., Ibrahim P., Gillespie J.B., Shahar R.A., McGuigan K.G. (2001). Effect of agitation, turbidity, aluminium foil reflectors and container volume on the inactivation efficiency of batch-process solar disinfectors. Water Res..

[109] Rijal G.K., Fujioka R.S. (2004). Use of reflectors to enhance the synergistic effects of solar heating and solar wavelengths to disinfect drinking water sources. Water Sci. Technol..

[110] Martín-Sómer M., Moreno-SanSegundo J., Álvarez-Fernández C., van Grieken R., Marugán J. (2021). High-performance low-cost solar collectors for water treatment fabricated with recycled materials, open-source hardware and 3d-printing technologies. Sci. Total Environ..

[111] Gueymard C.A. (2005). Interdisciplinary applications of a versatile spectral solar irradiance model: A review. Energy.

[112] National Center for Atmospheric Research Tropospheric Ultraviolet and Visible (TUV) Radiation Model. https://www2.acom.ucar.edu/modeling/tropospheric-ultraviolet-and-visible-tuv-radiation-model.

[113] Moreno-SanSegundo J., Giannakis S., Samoili S., Farinelli G., McGuigan K.G., Marugán J. (2021). SODIS potential: A novel parameter to assess the suitability of solar water disinfection worldwide. Chem. Eng. J..

[114] National Renewable Energy Laboratory, Solar Position Algorithm|NREL. http://www.nrel.gov/mide/solpos.spa.html.

[115] Lambert J.H. (1760). Photometria Sive de Mensura et Gradibus Luminis, Colorum et Umbrae.

[116] Beer A. (1852). Bestimmung der Absorption des rothen Lichts in farbigen Flüssigkeiten. Ann. Phys..

[117] Kirk J. (1994). Light and Photosynthesis in Aquatic Ecosystems.

[118] Boyd C.E. (2020). Solar radiation and water temperature. Water Quality.

[119] Brutsaert W. (1979). Heat and mass transfer to and from surfaces with dense vegetation or similar permeable roughness. Bound.-Layer Meteorol..

[120] Rutherford J.C., Blackett S., Blackett C., Saito L., Davies-Colley R.J. (1997). Predicting the effects of shade on water temperature in small streams. N. Z. J. Mar. Freshw. Res..

[121] Chick H. (1908). An investigation of the laws of disinfection. J. Hyg..

[122] Watson H.E. (1908). A note on the variation of the rate of disinfection with change in the concentration of the disinfectant. J. Hyg..

[123] Hom L.W. (1972). Kinetics of chlorine disinfection in an ecosystem. J. Sanit. Eng. Div..

[124] Chamberlin C.E., Mitchell R., Mitchell R. (1978). A decay model for enteric bacteria in natural waters. Water Polution Microbiology.

[125] Severin B.F., Suidan M.T., Engelbrecht R.S. (1982). Kinetic modeling of U.V. disinfection of water. Water Res..

[126] Castro-Alférez M., Polo-López M.I., Marugán J., Fernández-Ibáñez P. (2017). Mechanistic model of the *Escherichia coli* inactivation by solar disinfection based on the photo-generation of internal ROS and the photo-inactivation of enzymes: CAT and SOD. Chem. Eng. J..

[127] Silverman A.I., Nguyen M.T., Schilling I.E., Wenk J., Nelson K.L. (2015). Sunlight inactivation of viruses in open-water unit process treatment wetlands: Modeling endogenous and exogenous inactivation rates. Environ. Sci. Technol..

[128] Fisher M.B., Love D.C., Schuech R., Nelson K.L. (2011). Simulated sunlight action spectra for inactivation of MS2 and PRD1 bacteriophages in clear water. Environ. Sci. Technol..

[129] Silverman A.I., Nelson K.L. (2016). Modeling the endogenous sunlight inactivation rates of laboratory strain and Wastewater *E. coli* and *enterococci* using biological weighting functions. Environ. Sci. Technol..

[130] Lui G.Y., Roser D., Corkish R., Ashbolt N.J., Stuetz R. (2016). Point-of-use water disinfection using ultraviolet and visible light-emitting diodes. Sci. Total Environ..

[131] Vione D. (2021). The modelling of Surface-Water photoreactions made easier: Introducing the concept of ‘equivalent monochromatic wavelengths’. Water Res..

[132] Mancini J.L. (1978). Numerical estimates of coliform mortality rates under various conditions. Water Pollut. Control. Fed..

[133] Peleg M., Normand M.D., Corradini M.G. (2012). The Arrhenius equation revisited. Crit. Rev. Food Sci. Nutr..

[134] Ansys I. (2012). ANSYS FLUENT Theory Guide.

[135] Cassano A.E., Alfano O.M. (2000). Reaction engineering of suspended solid heterogeneous photocatalytic reactors. Catal. Today.

[136] Moreno J., Casado C., Marugán J. (2019). Improved discrete ordinate method for accurate simulation radiation transport using solar and LED light sources. Chem. Eng. Sci..

